# The broad range di- and tri-nucleotide exchanger SLC35B1 displays asymmetrical affinities for ATP transport across the ER membrane

**DOI:** 10.1016/j.jbc.2021.101537

**Published:** 2022-01-15

**Authors:** Pablo J. Schwarzbaum, Julieta Schachter, Luis M. Bredeston

**Affiliations:** Departamento de Química Biológica-IQUIFIB, Facultad de Farmacia y Bioquímica, Universidad de Buenos Aires-CONICET, CABA, Argentina

**Keywords:** ATP, nucleotide transporters, endoplasmic reticulum, membrane transporter structure, BiP, nucleotide metabolism, SLC35 transporters, AXER, nucleotide sugar transporters, chaperones, DDM, n-dodecyl-ß-D-maltopyranoside, ER, endoplasmic reticulum, hSLC35B1, human SLC35B1 isoform, NDP, dinucleotide, NMP, mononucleotide, NTP, trinucleotide, NST, nucleotide sugar transporter, PC, phosphatidylcholine, SLC35, solute carrier family 35, UDP-Glc, UDP-glucose, UGGT, UDP-glucose:glycoprotein glucosyltransferase

## Abstract

In eukaryotic cells, uptake of cytosolic ATP into the endoplasmic reticulum (ER) lumen is critical for the proper functioning of chaperone proteins. The human transport protein SLC35B1 was recently postulated to mediate ATP/ADP exchange in the ER; however, the underlying molecular mechanisms mediating ATP uptake are not completely understood. Here, we extensively characterized the transport kinetics of human SLC35B1 expressed in yeast that was purified and reconstituted into liposomes. Using [α^32^P]ATP uptake assays, we tested the nucleotide concentration dependence of ATP/ADP exchange activity on both sides of the membrane. We found that the apparent affinities of SLC35B1 for ATP/ADP on the internal face were approximately 13 times higher than those on the external side. Because SLC35B1-containing liposomes were preferentially inside-out oriented, these results suggest a low-affinity external site and a high-affinity internal site in the ER. Three different experimental approaches indicated that ATP/ADP exchange by SLC35B1 was not strict, and that other di- and tri-nucleotides could act as suitable counter-substrates for ATP, although mononucleotides and nucleotide sugars were not transported. Finally, bioinformatic analysis and site-directed mutagenesis identified that conserved residues K117 and K120 from transmembrane helix 4 and K277 from transmembrane helix 9 play critical roles in transport. The fact that SLC35B1 can promote ATP transport in exchange for ADP or UDP suggest a more direct coupling between ATP import requirements and the need for eliminating ADP and UDP, which are generated as side products of reactions taking place in the ER-lumen.

The supply of specific solutes is essential for protein folding and maturation taking place in the lumen of the endoplasmic reticulum (ER) (1). Uptake of ATP into ER is required for molecular chaperones BiP and other heat shock proteins whose functioning is coupled to binding and hydrolysis of ATP, generating ADP (2, 3). On the other hand, uptake of UDP-glucose (UDP-Glc) is necessary to promote the UDP-glucose:glycoprotein glucosyltransferase (UGGT) dependent transfer of glucose to noncompletely folded glycoproteins, generating the accumulation of intraluminal UDP. Repeated association of the mono-glucosylated glycoproteins with calnexin or calreticulin chaperones and dissociation after the action of α-glucosidase II, constitutes an essential cycle in the maturation of glycoproteins to reach the native form (4). ATP and UDP-Glc are not synthesized in the ER and must therefore be transported from the cytosol (5). The molecular mechanisms responsible for ER uptake of these compounds remained an enigma for a long time (6).

In analogy with transport occurring in the Golgi apparatus, it was initially postulated that a nucleotide sugar transporter (NST) belonging to the solute carrier family 35 (SLC35) could transport UDP-Glc from the cytosol to the ER lumen by a UDP-Glc/UMP antiport mechanism (5, 7). Several reports associated the hut1 membrane proteins of yeast (8) and worms (9) or its homolog AtUTr1 of plants (10) with ER stress and activation of the unfolded protein response, and postulated them as candidates for the transport of UDP-Glc to the ER. However, subsequent studies in yeast demonstrated that conversion of UDP to UMP by a diphosphonucleotidase system was absent in the ER (11, 12). Furthermore, UGGT assayed *in vivo* was active in yeast mutants where putative NST members were deleted, including hut1 (13). These results cast doubt on the relationship of hut1 with the entry of UDP-Glc into the ER lumen, suggesting that another mechanism independent of the NST family could be involved (13).

Regarding the transport of ATP to the ER, the information has been scarce. Early work by Hirschberg's group showed the transport of ATP into ER-derived microsomes from canine pancreas and rat liver or ER-derived proteoliposomes (5, 14, 15). Although ATP translocation was shown to be saturable and protein mediated, identification of the transporter remained elusive, with the exception of the Arabidopsis carrier ER-ANT1, with homologs restricted only to plants (16, 17) and Sac1p, an unconfirmed yeast transporter (18, 19).

A recent report combining bioinformatics tools and an experimental approach begun to clarify the state of confusion in relation to the transport of UDP-Glc and ATP in the ER. Klein *et al.* (20) showed that the human SLC35B1 transporter (hSLC35B1), homologous to hut1 (8, 9, 10), is responsible for the entry of ATP into the ER. Assays using intact *Escherichia coli* expressing hSLC35B1 in the inner membrane showed the uptake of [α^32^P]ATP or [α^32^P]ADP. Furthermore, transport assays, after solubilization with detergents of membranes expressing hSLC35B1 and reconstitution into liposomes, allowed these authors to propose an ATP/ADP counter-exchange mechanism. Combination of antibody detection of hSLC35B1, knockdown, and live cell imaging of ER-ATP levels support the idea that hSLC35B1 represent an important ATP/ADP exchanger of the ER membrane (20). More recently, a study of ATP dynamics using various mammalian cells lines showed that SLC35B1 mediates the mitochondria supply of ATP to ER by a mechanism regulated by cytosolic calcium (21). These findings resignified previous results showing ER stress due to the decrease in protein expression as a consequence of the knockout of putative genes homologous to hSLC35B1 like CeHUT1 in *Caenorhabditis elegans* (9), SpHUT1 in *Schizosaccharomyces pombe* (8, 13), or AtUTr3 in *Arabidopsis thaliana* (10). In the absence of information on their specificity, these transporters were erroneously thought to be involved in UDP-Glc import.

In spite of this progress, whether SCL35B1 behaves as a strict ATP/ADP antiporter, as occurred in the well characterized mitochondrial ADP/ATP carrier from the unrelated SLC25 family (22, 23), remains to be inquired.

Accordingly, in this work, we carried out a detailed characterization of hSLC35B1 expressed in yeast, purified, and reconstituted in PC-liposomes. Uptake studies using [α^32^P]ATP showed that hSLC35B1-containing liposomes acts as an antiport mechanism enabling the exchange of di- and tri-nucleotides, including ATP/ADP exchange, with asymmetrical apparent affinities at both sides of the membrane. Furthermore, combination of bioinformatics and mutational analysis identified three key residues involved in hSLC35B1 function. Our findings allowed to deepen the understanding of the role of SLC35B1 as an ATP import mediator of ER, and as a broad-range antiporter for nucleotides.

## Results

### Import [α^32^P]ATP of purified hSLC35B1 after reconstitution into liposomes loaded with ADP

**Figure 1:**
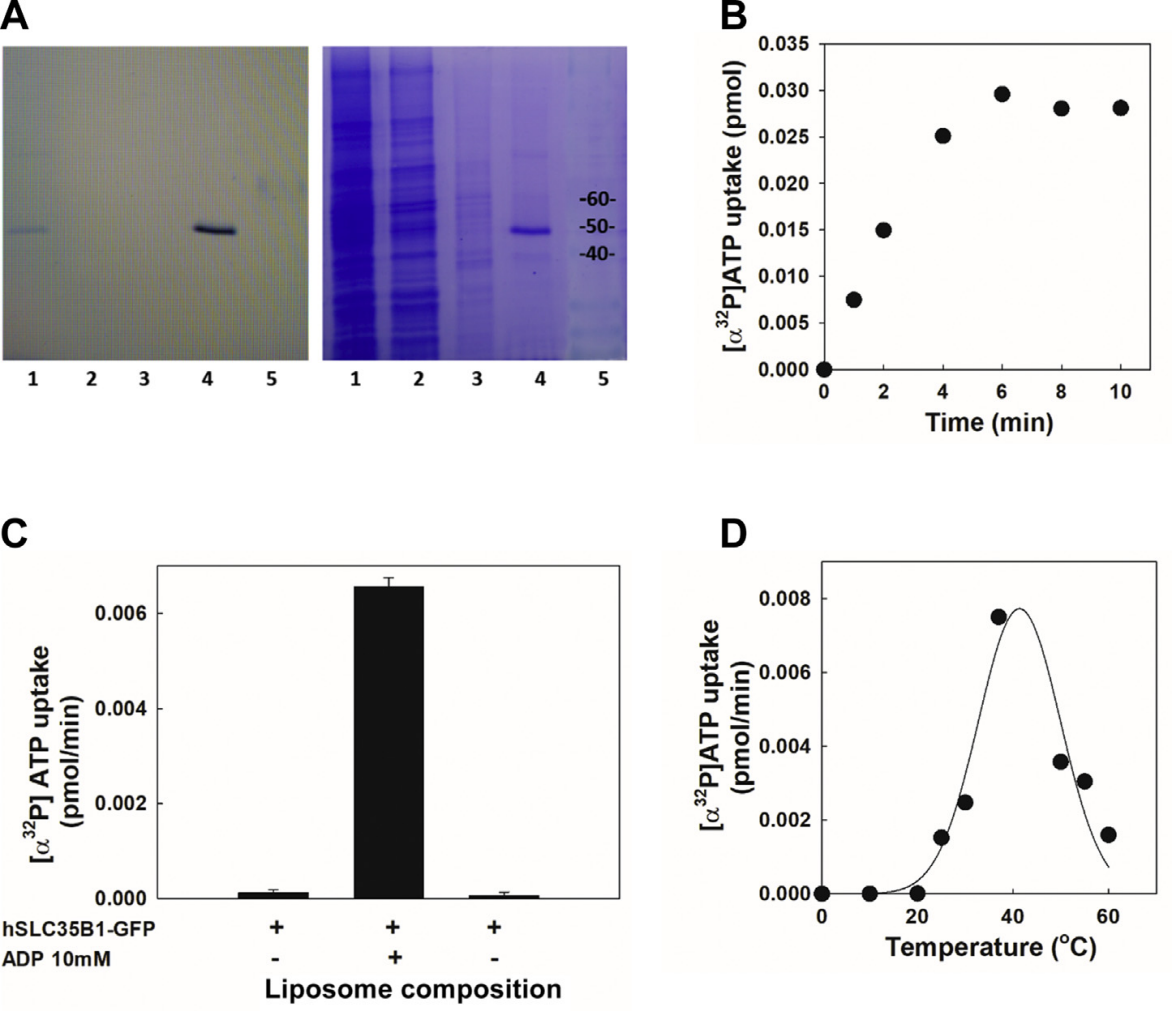
**Functional properties of purified hSLC35B1-GFP inserted in PC-liposomes.***A*, SDS-PAGE analysis of hSLC35B1-GFP purification procedure. Gel fluorescence (*left*) and Coomassie blue staining (*right*) of solubilized membranes (lane 1), flow through (lane 2), sample washed with 30 mM imidazole buffer (lane 3), sample eluted with 200 mM imidazole buffer (lane 4), and molecular weight markers (lane 5). *B*, time dependence of [α^32^P]ATP uptake (at 0.1 μM) in hSLC35B1-liposomes (loaded with ADP 10 mM) incubated at the indicated times at 37 °C. *C*, dependence of PC-liposomes composition on ATP uptake incubated in a media containing 0.1 μM [α^32^P]ATP for 1 min at 37 °C. The bars represent the mean values + SEM (n = 3). *D*, effect of temperature on [α^32^P]ATP uptake. hSLC35B1-liposomes were incubated for 2 min with 0.1 μM [α^32^P]ATP at the indicated temperatures. The reaction was stopped, and the incorporated [α^32^P]ATP to hSLC35B1-liposomes was measured as indicated in Experimental procedures. hSLC35B1, human SLC35B1 isoform; PC, phosphatidylcholine.

Human SLC35B1 isoform 1 was cloned in plasmid p426GFP and expressed in *Saccharomyces cerevisiae* as a C-terminal GFP fusion protein (hSLC35B1-GFP). After membrane isolation and n-dodecyl-ß-D-maltopyranoside (DDM)-detergent solubilization, hSLC35B1-GFP was purified by NTA-Ni as described in Experimental procedures. The fractions of the purification procedure were analyzed by SDS-PAGE and revealed by gel fluorescence and Coomassie blue staining (Fig. 1*A*, left and right). After column washing with a buffer containing 30 mM imidazole (lane 3, left and right), the fraction eluted in the same buffer containing 200 mM Imidazole (lane 4, left and right) showed a main band of approximately 50 kDa, smaller than the predicted 65 kDa value corresponding to the fusion hSLC35B1-GFP. This is probably because of anomalous mobility of the fusion protein, as we and others observed for other membrane proteins, including members of the NST family (24, 25). To assay transport activity, purified hSLC35B1-GFP was reconstituted in PC-liposomes (hSLC35B1-liposomes). In a first series of experiments, hSLC35B1-liposomes were loaded with 10 mM ADP and uptake of 0.1 μM [α^32^P]ATP was tested. An illustrative time dependence of [α^32^P]ATP transport is shown (Fig. 1*B*). In addition, similar experiments were performed using ADP-loaded liposomes lacking hSLC35B1-GFP and hSLC35B1-liposomes lacking a loading nucleotide. The results showed that hSLC35B1-GFP is kinetically active, and that only liposomes containing the transporter protein and loaded with ADP exhibited the capacity to import [α^32^P]ATP (Fig. 1*C*), since the absence of either hSLC35B1-GFP or internal ADP showed virtually no signal. These results confirmed a previous study showing ATP/ADP exchange in proteoliposomes prepared from solubilized total membrane proteins of bacteria expressing hSLC35B1 (20). The temperature dependence of [α^32^P]ATP uptake was tested by incubating hSLC35B1-liposomes at 0 to 60 °C (Fig. 1*D*), showing a temperature-dependent increment of [α^32^P]ATP uptake to a maximum at approximately 37 °C, followed by a decrease at higher temperatures, probably because of protein denaturation.

### Concentration dependence of nucleotides on hSLC35B1 transport differs on both sides of the liposome membrane

**Figure 2:**
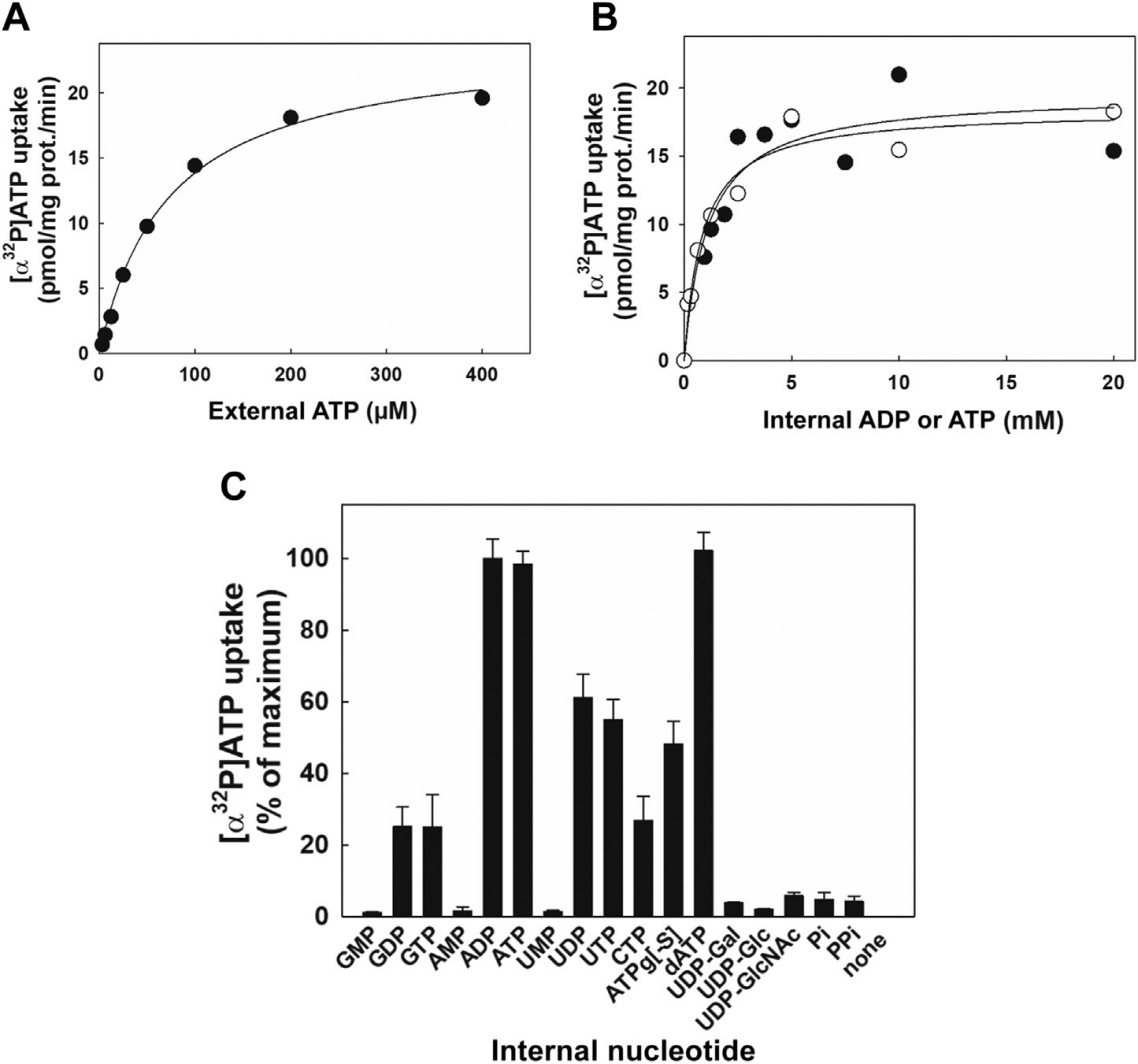
**Concentration and nucleotide specificity dependence; external and internal nucleotide concentration effect on hSLC35B1 transport activity.***A*, dependence of [α^32^P]ATP uptake on external ATP concentration. hSLC35B1-liposomes were loaded with 10 mM ADP and incubated with the indicated [α^32^P]ATP concentrations for 1 min at 37 °C. *B*, dependence of [α^32^P]ATP uptake on internal concentrations of ADP (●) or ATP (○). hSLC35B1-liposomes were loaded with the indicated ADP or ATP concentrations and incubated with 200 μM [α^32^P]ATP for 1 min at 37 °C. In all cases, the reaction was stopped and the incorporated [α^32^P]ATP to hSLC35B1-liposomes was measured. The *continuous lines* represent the fitting of a single hyperbolic function to experimental data. The best-fit parameter values were Vmax: 23.91 ± 0.82 pmol/mg prot./min, K_0.5(external ATP)_: 72.45 ± 7.00 μM (*A*) and Vmax: 19.58 ± 1.86 pmol/mg prot./min, K_0.5(internal ADP)_: 1.09 ± 0.43 mM and Vmax: 18.38 ± 0.96 pmol/mg prot./min; K_0.5(internal ATP)_: 0.84 ± 0.18 mM (*B*). *C*, effect of internal nucleotides on [α^32^P]ATP uptake by hSLC35B1-liposomes. The hSLC35B1-liposomes were loaded with 10 mM of the indicated nucleotides, nucleotide sugars, phosphate (Pi) or pyrophosphate (PPi), and incubated with 200 μM [α^32^P]ATP for 1 min at 37 °C. The reaction was stopped and the incorporated [α^32^P]ATP to hSLC35B1-liposomes was measured. A value of 14.77 ± 0.79 pmol/mg prot./min, corresponding to the uptake in hSLC35B1-liposomes loaded with ADP, was considered 100% of [α^32^P]ATP uptake activity. The bars represent the mean + SEM (n = 3). hSLC35B1, human SLC35B1 isoform.

Next, we studied the dependence of the [α^32^P]ATP uptake on external ATP concentration and internal concentrations of either ADP or ATP. Human SLC35B1-liposomes were initially loaded with 10 mM ADP and incubated with variable [α^32^P]ATP concentrations (1–400 μM). The rates of [α^32^P]ATP uptake followed a hyperbolic-like dependence with external ATP (Fig. 2*A*), yielding an apparent affinity value (K_0.5(external ATP)_) of 72.45 ± 7.00 μM. A similar assay using [ɤ^32^P]ATP instead of [α^32^P]ATP yielded a similar K_0.5(external ATP)_ (64.67 ± 8.00 μM; see Fig. S1). These results strongly suggest that the purified preparation is free of contaminating hydrolases or kinases capable of degrading ATP and, therefore, both radionuclides can be used interchangeably to test hSLC35B1 transport properties.

When hSLC35B1-liposomes loaded with increasing concentrations of either internal ADP or ATP (0–20 mM) were incubated in the presence of 200 μM [α^32^P]ATP, uptake showed a hyperbolic-like behavior (Fig. 2*B*) yielding K_0.5(internal ADP)_ = 1.10 ± 0.43 mM and K_0.5(internal ATP)_ = 0.84 ± 0.18 mM. These values were 13-fold higher than that of K_0.5(external ATP)_ (Fig. 2*A*). The observed differences in the apparent affinity values obtained at both sides of the membrane liposomes suggest an asymmetry in the requirements of nucleotide concentrations to activate hSLC35B1 transport. As a control of the actual intravesicular nucleotides concentrations in proteoliposome preparations, we verified that the loading concentration of ATP closely matches the concentration of ATP trapped in the lumen of proteoliposomes. (See Fig. S2*A*). On the other hand, a digestion assay using TEV protease showed that most of the TEV cleavage sequence linking GFP with the C-terminal part of hSCL35B1 is inaccessible in intact proteoliposomes, whereas disruption with Triton X-100 allowed GFP release (Fig. S2*B*). These results suggest an insertion of SCL35B1-GFP with the GFP-associated portion oriented to the luminal side of the liposome.

### Import of ATP by hSLC35B1 can be driven by luminal purine or pyrimidine derived di- or tri-nucleotides

We next tested the possibility that nucleotides other than ATP or ADP can promote counter-transport, thus allowing [α^32^P]ATP uptake. This is relevant in the light of results of ligand competition assays suggesting that hSLC35B1 functions as a strict antiport of ATP and ADP (20). Then, hSLC35B1-liposomes were loaded with 10 mM of various nucleotides, nucleotide sugars, inorganic phosphate (Pi), or pyrophosphate (PPi), and the uptake of 200 μM [α^32^P]ATP was tested (Fig. 2*C*). The results showed that initial rates of [α^32^P]ATP uptake in the presence of internal ADP or ATP were 14.77 or 13.52 pmol/mg prot./min, respectively. These values were considered maximal, as they could only be replicated by dATP (Fig. 2*C*).

Compared to maximal values, [α^32^P]ATP uptake of UTP- or UDP-loaded hSLC35B1-liposomes was 55 to 60%, whereas in the presence of internal GDP, GTP, or CTP, uptake values were reduced to 20 to 30%. Finally, hSLC35B1-liposomes loaded with nucleoside monophosphates (GMP, AMP, or UMP), UDP-sugars (-galactose, -glucose or -N-acetylglucosamine), Pi, or PPi were not able to promote [α^32^P]ATP uptake (Fig. 2*C*).

Transport rates observed in the presence of di- or tri-nucleotides (NDP or NTP), as compared to mononucleotides (NMP), suggest the requirement of at least two phosphate groups of the nucleotides to activate [α^32^P]ATP transport. On the other hand, UTP and UDP could act as suitable nucleotides promoting counter-transport of hSLC35B1. To complement these findings, Fig. S3 shows similar experiments to those of Figure 2, *A* and *B* but using internal UDP. It can be seen that [α^32^P]ATP uptake followed a hyperbolic function of UDP with K_0.5(internal UDP)_ = 6.03 ± 1.2 mM, that is, a low apparent affinity value (Fig. S3*A*). Moreover, using 20 mM UDP-loaded hSLC35B1-liposomes, K_0.5_ for [α^32^P]ATP amounted to 34 μM (Fig. S3*B*), that is, a value similar to that obtained using 10 mM internal ADP (Fig. 2*A*).

### Addition of external di- and tri-nucleotides impairs ATP import to hSLC35B1-liposomes in a concentration-dependent manner

The results above imply that internal NDP and NTP are required in millimolar concentrations inside the hSLC35B1-liposomes to promote [α^32^P]ATP import. Assuming a transport model where hSLC35B1 exposes a single site alternatively to each side of the liposome membrane, then these nucleotides, when applied externally, should compete, and inhibit ATP import.

**Figure 3:**
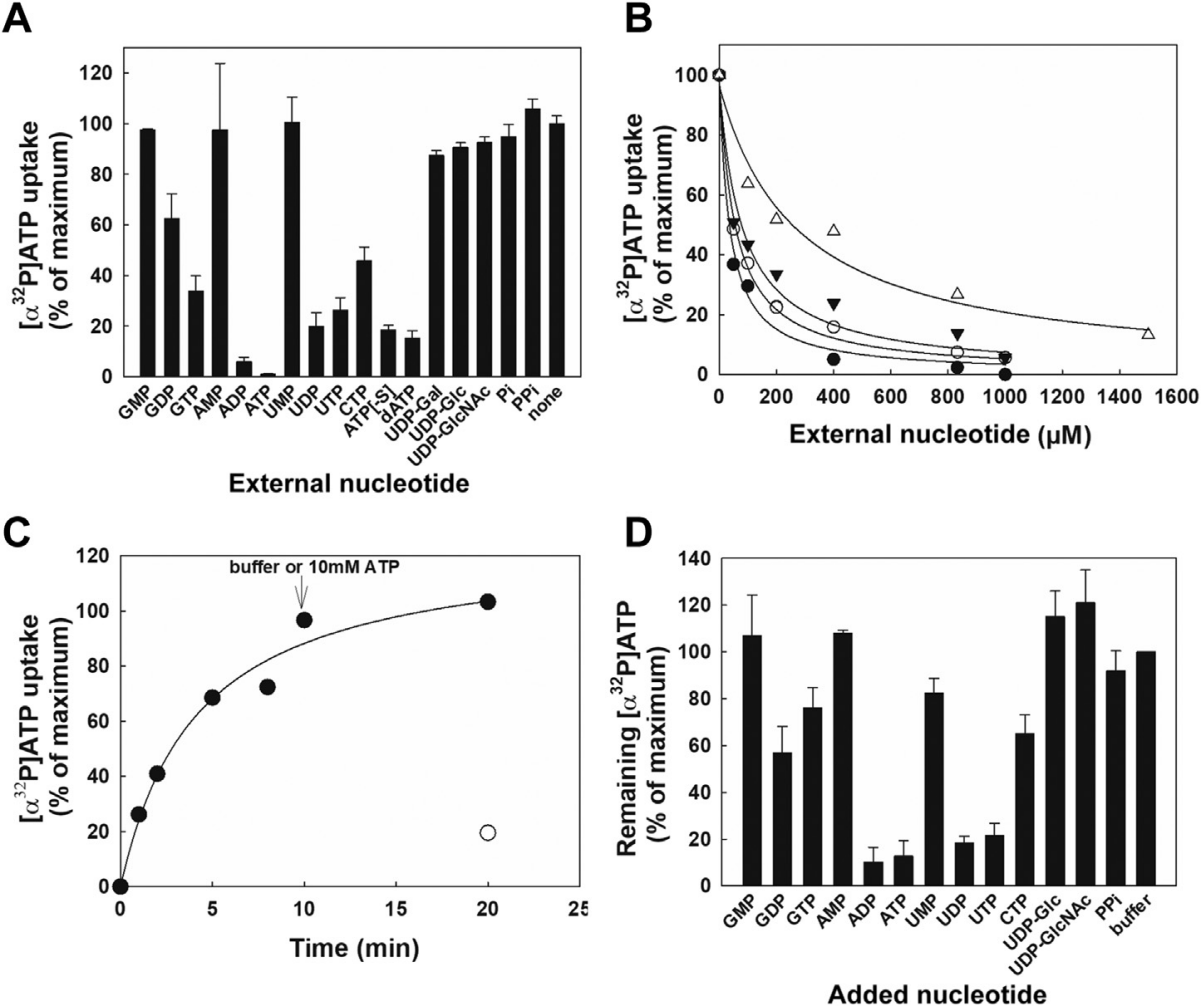
**Effect of external nucleotides on** [**α**^**32**^**P]ATP uptake and efflux by hSLC35B1-liposomes.***A*, competition assay. The effects of the indicated nucleotides, nucleotide sugars (NS), phosphate (Pi), or pyrophosphate (PPi) on [α^32^P]ATP uptake was assayed in hSLC35B1-liposomes loaded with 10 mM of ADP. hSLC35B1-liposomes were incubated with 0.1 μM [α^32^P]ATP for 1 min at 37 °C in the presence of buffer or 1 mM of the indicated nucleotides, NS, Pi or PPi. The bars represent the mean values + SEM (n = 3). *B*, effect of nucleotide concentration on [α^32^P]ATP uptake. hSLC35B1-liposomes were incubated with 0.1 μM [α^32^P]ATP for 1 min at 37 °C in the presence of the indicated concentrations of ATP(●), ADP (○), UDP (▼), or GTP (△). After running incubation, the reaction was stopped and the incorporated [α^32^P]ATP to hSLC35B1-liposomes measured, as indicated in Experimental procedures. A value of 0.0032 pmol/mg prot./min, corresponding to [α^32^P]ATP uptake in the absence of added ligands was taken as 100% of [α^32^P]ATP uptake activity (n = 3). The continuous lines represent the fitting of a single hyperbolic decay function to experimental data. The best-fit parameter values were: Vmax: 99.53 ± 4.77%, Ki (ATP): 35.98 ± 5.58 μM; Vmax: 99.21 ± 2.56%, Ki (ADP): 56.05 ± 4.37 μM; Vmax: 96.86 ± 6.25%, Ki (UDP): 82.98 ± 16.03 μM; and Vmax: 96.44 ± 5.91%, Ki (GTP): 275.04 ± 53.93 μM. *C*, nucleotide mediated efflux of [α^32^P]ATP from hSLC35B1-liposomes. SLC35B1-liposomes loaded with 10 mM of ATP were incubated with 200 μM [α^32^P]ATP for the indicated times at 37 °C, and the time course of [α^32^P]ATP uptake was measured. At 10 min, buffer (●) or 10 mM ATP (○) were added to the reaction media and incubated for additional 10 min, after which the reaction was stopped and the remaining [α^32^P]ATP associated with hSLC35B1-liposomes was measured. *D*, experiments run similarly to (*C*) but adding 10 mM of different unlabeled nucleotides or buffer. A value of 31 pmol[α^32^P]ATP/mg prot., after an incubation of 20 min in the absence of ligands, was considered 100% of the remaining [α^32^P]ATP. The bars represent the mean values + SEM (n = 3). hSLC35B1, human SLC35B1 isoform.

Accordingly, hSLC35B1-liposomes were loaded with 10 mM of ADP, and the uptake of 0.1 μM [α^32^P]ATP was monitored at 37 °C, in the absence and presence of 1 mM of unlabeled nucleotides. Figure 3*A* shows that the inhibition of [α^32^P]ATP uptake was maximal with external ATP and ADP (>90%), with ATP analogs dATP and ATPɣ[-S] inhibiting >85%. The addition of UDP, UTP, or GTP were slightly less effective (70–85%), whereas GDP or CTP caused a transport reduction of 50 to 60%. No effect was observed with NMPs (GMP, AMP, or UMP), UDP-sugars (-Glucose, -Galactose or -N-Acetylglucosamine), Pi, or PPi, all of which displayed similar values to control experiments run in the absence of added ligands.

Next, the concentration dependence of selected nucleotides on [α^32^P]ATP uptake was studied incubating the hSLC35B1-liposomes with 0.1 μM [α^32^P]ATP for 1 min at 37 °C and the indicated concentrations of ATP, ADP, UDP, or GTP (Fig. 3*B*). All the nucleotides tested showed a concentration-dependent inhibitory effect on [α^32^P]ATP uptake. Inhibition was well described by a hyperbolic decay with apparent Ki in the micromolar range, with values being ATP ≈ ADP > UDP > GTP (Fig. 3*B*).

### Maximum efflux of [α^32^P]ATP is driven by external di- or tri-nucleotides of adenosine and uridine

To complete the characterization of nucleotide specificity, and the capacity of various nucleotides to exchange with imported [α^32^P]ATP from the external side, an efflux assay was performed. The hSLC35B1-liposomes loaded with 10 mM of ATP were incubated with 200 μM [α^32^P]ATP at 37 °C and the time dependence of [α^32^P]ATP uptake was monitored. After 10 min, a buffer or 10 mM ATP (or various nucleotides) were added and the assay was run for additional 10 min (Fig. 3, *C* and *D*). Then, residual [α^32^P]ATP inside liposomes was measured at t = 20 min. The rationale behind this assay is that the activation of ATP efflux should reduce residual values of [α^32^P]ATP inside hSLC35B1-liposomes. Compared to values obtained after addition of buffer, residual [α^32^P]ATP was decreased 80 to 90% in the presence of ADP, ATP, UDP, or UTP and 30 to 35% with GDP, GTP, or CTP (Fig. 3*D*), whereas in the presence of external NMPs, nucleotide sugars, or PPi, nonsignificant changes were detected. These results suggest that, under the experimental conditions, ADP, ATP, UDP, or UTP were more effective in activating [α^32^P]ATP efflux, with relatively lower effects were induced by GDP, GTP, or CTP.

Up to here, the results show that hSLC35B1-liposomes displays two very distinct apparent affinities for transport: a high apparent affinity for ATP import and a relatively low apparent affinity for nucleotides acting on the internal side. We also found that di- and tri-nucleotides other than ATP and ADP, notably UTP and UDP, can be transported by hSLC35B1. These nucleotides can act as suitable cotransport partners of ATP, and at the same time compete with ATP for the external binding site. Nucleoside monophosphates and nucleotide sugars tested neither compete nor were they transported.

### Mutation analysis of evolutionary conserved residues

**Figure 4:**
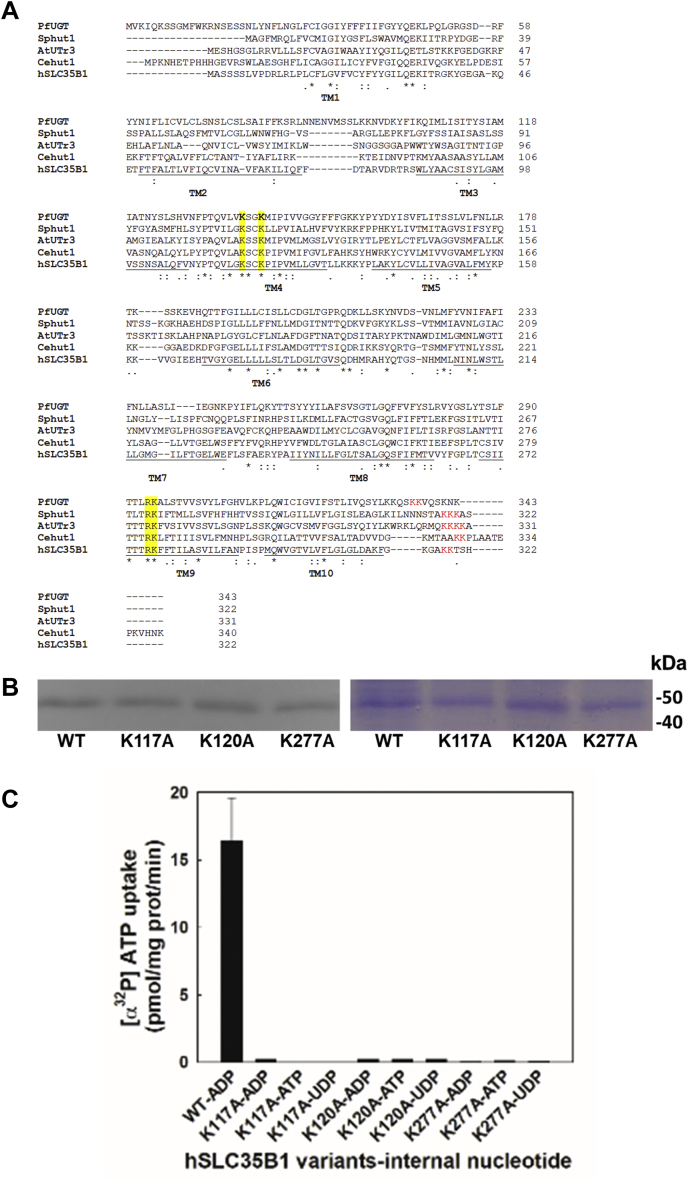
**Mutational analysis of evolutionary conserved residues.***A*, alignment of hSLC35B1 and putative orthologs from evolutionary distant organism: *A. thaliana* (AtUTr3), *S. pombe* (Sphut1), and *C. elegans* (CeHut1). Conserved charged residues were labeled in *yellow*, and the C-terminal ER retention motif was labeled in *red*. *B*, proteins purified from yeast membranes expressing WT, K117A, K120A, or K277A variants were separated by SDS-PAGE and GFP fusions visualized by gel fluorescence and CB staining. *C*, [α^32^P]ATP uptake activity of purified hSLC35B1-GFP variants inserted in liposomes loaded with 10 mM of ADP, ATP, or UDP, and incubated with 200 μM [α^32^P]ATP for 1 min at 37 °C. The bars represent the mean values + SEM (n = 3). ER, endoplasmic reticulum; hSLC35B1, human SLC35B1 isoform.

To identify residues on the transmembrane helices of the hSLC35B1 that could potentially interact with the negative charged nucleotides characterized above, bioinformatics analysis was performed. An alignment of hSLC35B1 and putative orthologous sequences from evolutionarily distant organisms (*A. thaliana*, *S. pombe*, and *C. elegans*) showed four conserved charged residues (Fig. 4*A*) in the transmembrane segments TM4 (K117 and K120) and TM9 (R276 and K277), which proved interesting for mutational analysis. After obtaining cDNA corresponding to the alanine substitution of each of these residues and cloning in p426GFP plasmid, the variants were expressed in yeast as GFP fusion proteins. All variants expressed at similar levels to that of WT, as judged by fluorescence intensities of whole cells, except for the R276A variant that was not detectable. Then, membranes were isolated and the expressed variants K117A, K120A, and K277A were purified after solubilization with DDM, as was performed previously for WT-hSLC35B1 (Figs. 4*B* and S4). To test the effect of the substitution on transport activity, the corresponding purified variants were reconstituted in liposomes loaded with 10 mM of ADP, ATP, or UDP and incubated with 200 μM [α^32^P]ATP for 1 min at 37 °C (Fig. 4*C*). Substitutions K117A, K120A and K277A completely abolished ATP uptake under all the conditions tested, thus suggesting that the selected residues could be relevant during the initial recognition of the binding site and/or the transport of nucleotides.

**Figure 5:**
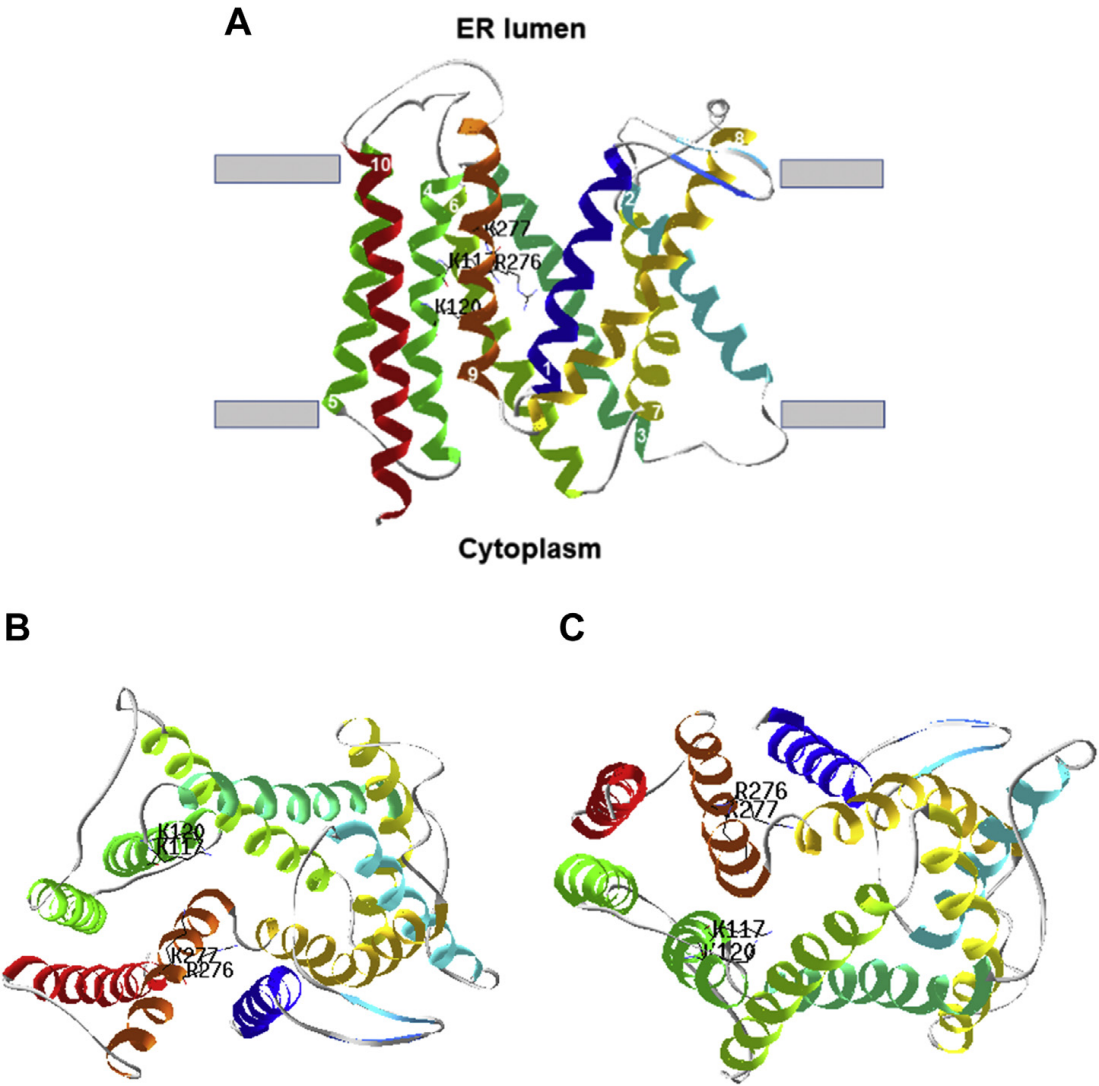
**Homology-based model of hSLC35B1.** A 3D-model of hSLC35B1 was generated (confidence 100%, coverage 92%) as described under Experimental procedures. Ribbon representation of the structure, viewed from the plane of the membrane (*A*), luminal side (*B*), and cytosolic side (*C*). TM4 (containing residues K117 and K120) and TM9 (containing residues R276 and K277) were labeled in *green* and *orange*, respectively. *Numbers* in *white* indicated the N-terminal of each transmembrane helix. hSLC35B1, human SLC35B1 isoform.

Finally, a model of hSLC35B1 was built based on the experimental resolved 3D-structure of Vrg4 protein, a yeast GDP-mannose transporter (26). The results of Figure 5 illustrate the characteristic fold of 10-TM of the hSLC35B1 model, described for diverse members of the Drug Metabolite Transporter superfamily, previously solved by X-ray or cryo-ME (26, 27, 28, 29, 30, 31). This fold consists of two inverted repeats, the N-(TM1-5) and -C (TM6-10) halves. A detailed inspection of the model predicted that residues K117 and K120 (on TM4) and R276 and K277 (on TM9) of hSLC35B1 are located on the middle of the membrane, as part of a central cavity, which is compatible with the formation of a binding site than can interact with negative charged nucleotides.

## Discussion

Human SLC35B1 was recently reported to facilitate the counter-transport of ATP/ADP at the ER membrane (20). Because other nucleotides and nucleotide sugars participate in key biochemical reactions within the ER, the main goal of this study was to test and kinetically characterize the capacity of hSLC35B1 to promote the transport of a range of nucleotides and derived molecules.

In the first series of experiments, the uptake of [α^32^P]ATP into hSLC35B1-liposomes was shown to require ADP as counter-substrate (Fig. 1*C*). The transport displayed an optimal temperature around 37 °C (Fig. 1*D*), whereas prolonged incubation with [α^32^P]ATP led to isotopic equilibration in both compartments (Fig. 1*B*).

Kinetic characterization of the transporter using high internal ADP concentration shows saturable kinetics of [α^32^P]-ATP uptake, with apparent K_0.5_ ≈ 70 μM (Fig. 2*A*).

When ATP transport was tested at variable internal concentrations of ATP or ADP, the nucleotide apparent affinities on the internal side of the liposomes lay within the millimolar range (*i.e.*, K_0.5_ = 0.8–1 mM using internal ATP or ADP) (Fig. 2*B*).

Interestingly, this feature was not exclusive to adenine nucleotides, because [α^32^P]ATP uptake at variable internal UDP concentrations also occurred with low apparent affinity (K_0.5(internal UDP)_ = 6 mM). In fact, UTP and UDP, and to a lesser extent GTP and GDP, behave as suitable counter-substrates promoting transport (Fig. 2*C*). Considering the preferential inside-out orientation of our hSLC35B1-liposome preparation, these results suggests that *in vivo* the transporter might work in ER with low apparent affinity on the cytosolic side and high apparent affinity on the intraluminal side of ER. In most cell types, cytosolic ATP is millimolar, that is, in the range of the internal K_0.5_ (internal to the inside out hSLC35B1iposomes and external to the ER) found in proteoliposomes. Although speculative, this means that the transporter *in vivo* may promote ER uptake of ATP at submaximal speeds and may change ATP transport rates according to the effective ATP concentration at the ER surface.

Having kinetically characterized the basic exchange modes of hSCL35B1, we further studied the requirements of potential ligands for binding and transport. Chase experiments using [α^32^P]ATP and an excess of unlabeled ligands clearly showed that NDPs and NTPs, but not NMPs or UDP-sugars, were suitable competing ligands at the external site, promoting strong cis-inhibition of [α^32^P]ATP uptake (Fig. 3*A*). Various ER-related substrates tested showed a concentration-dependent inhibition of transport, with apparent affinities being ATP ≈ ADP > UDP > GTP (Fig. 3*B*). The high inhibitory effect of dATP and ATPɣ[-S] suggests that replacing a hydroxyl group by hydrogen at the 2′ position of the ribose or a =O group at ɣ-phosphate by =S is not crucial for the recognition of the hSLC35B1-binding site. On the other hand, the competitive inhibitory effect of UDP or UTP and the lack of effect of UDP-sugars suggest an impediment to accommodate a sugar (instead of a Pi) at the external binding site of hSLC35B1.

To assess which competing ligands could be transported, [α^32^P]ATP efflux experiments were made. Based on the antiport nature of the proposed transport mechanism (5, 20), these nucleotides need to be taken up in order for ATP efflux to be triggered. In line with chase experiments, NDPs and NTPs were effective in activating ATP efflux, with ATP, ADP, UDP, and UTP being more effective than GTP and CTP (Fig. 3*D*).

### Broad exchange of nucleotides at the ER

The above analysis reveals various key features of hSLC35B1. Provided that a counter-transport substrate is present to allow transport, ATP supply is relevant considering that ER is unable to synthetize ATP and the requirement of intraluminal ATP for protein folding (32).

Among several intraluminal ATP-binding proteins, BiP is very abundant in the ER, displays high affinity for ATP (33, 34) and has been postulated to be one of the most important ATP consumers (3). Moreover, protein misfolding has been shown to increase ATP uptake (21). Thus, hSLC35B1 may supply ATP to BiP, thereby promoting the quality control of protein folding and the unfolded protein response (6, 32).

Regarding the capacity of hSCL35B1 to transport NDPs, notably ADP and UDP, previous reports suggested that NDPs may be metabolized in the ER, rather than exported. In this respect, hSLC35D1, a proposed ER-transporter (35), facilitates the uptake of UDP-sugars in exchange for UMP (36, 37). Luminal UDP-sugars can then act as substrates for glycosylation reactions, where UDP is a by-product. If unrestrained, continuous accumulation of intraluminal UDP may inhibit glycosyltransferases (38, 39). In this context, UDP was proposed to be converted to UMP by an ER-nucleoside diphosphatase (specific for UDP, GDP, and IDP, but not for ADP) (38, 40), followed by UMP exit, in analogy to the antiport mechanism of SLC35 members present in the Golgi apparatus (5).

However in yeasts, where SLC35D1 homologs are absent, deletion of the nucleoside diphosphatase activities of the secretory pathway (11, 12) or of the NSTs with retention signal in the ER (13) did not interrupt the *in vivo* activity of UGGT, suggesting that UDP-Glc entry to the ER is driven by a mechanism independent of the conversion of UDP into UMP and/or independent of transporters of the SLC35 family. Thus, our results provide an alternative mechanism to remove intraluminal UDP by efflux, rather than enzymatic degradation. In line with this idea, the decrease in UDP-GlcA-dependent glucuronosyltransferase activity in the ER lumen after the knockdown of SLC35B1 (41) may be partly because of defective UDP export.

Similarly, because SCL35B1 promotes ADP efflux, it may remove unnecessary ADP from the ER lumen, while providing ADP for glycolytic ATP synthesis in the cytosol and/or for ADP/ATP exchange by the mitochondrial carrier (23, 42).

Another mechanism to control transport rates relates to the concentration of hSCL35B1 itself, which is assumed to be very low (low nanomolar in HeLa, see refs (20)). Nevertheless, the concentration of the transporter, and therefore its macroscopic maximal activity may be dynamic, because knockdown of hSCL35B1 in HeLa cells led to a decrease of intraluminal ATP (20, 21), whereas stress conditions caused increased expression of the SCL35B1 gene in a wide variety of organisms expressing ortholog variants (9, 43).

Our results show that hSCL35B1 activity also mediated the transport of GTP and CTP, which is lower compared to that of uridine and adenine NDP and NTPs, but nevertheless significant.

### Role of charged residues on transport mechanism

Finally, mutational analysis was carried out to seek for key residues involved in nucleotide transport. Sequence alignment and topology prediction of SLC35B1-4 members showed that TM4 and TM9 transmembrane helices contain charged residues surrounded by hydrophobic residues (Fig. S5), suggesting that they could participate in the interaction with transported nucleotides or nucleotides derivatives. The KXXK motif is present in the TM4 of SLC35B1-B3 sequences, though SLC35B4 showed a different KXXX motif. On the other hand, the RK motif of TM9 is conserved in all members but SLC35B2, which exhibits a RQ motif. Except for SLC35B3, all analyzed sequences contain the c-terminal ER-retention motif XKKX after TM10 (Fig. S5). Then, KXXK and RK motifs on TM4 and TM9, respectively, and the XKKX retention signal at the c-terminus appear to be a characteristic feature only in the hSLC35B1 subfamily, a feature being evolutionarily conserved from yeast to mammalians (Fig. 4*A*). Interestingly, our results showed that replacing lysine residues K117, K120, or K277 by alanine fully abolished hSCL35B1 transport (Fig. 4*C*). The sequences of most other members of the SLC35 family characterized as NST show at least one positive charged residue in the TM4 or TM9 (25). Notably, the same occurs in other members that make up the DMT superfamily such as GsGPT, a triosephosphate/phosphate translocator of the inner membrane of chloroplasts, whose resolved 3D structure in the presence of substrates shows that the oxygens of the only phosphate group of 3-phosphoglycerate or the inorganic phosphate are coordinated by the K204 of TM4 and by the residues K362 and R363 of TM9, equivalent to residues K117, R276, and K277 of hSLC35B1. Thus, the lack of activity of hSCL35B1 mutants K117A, K120A, and K277A suggests that these residues, and the conserved R276 (Fig. 5), play a key role during the transport process, probably in the coordination of at least two phosphate groups present in the NDPs or NTPs. One might speculate that NMP might not be able to fully engage with all contact points at the active site, rendering WT hSCL35B1 unable to trigger NMPs transport, as our transport studies show. A similar mechanism was postulated for the unrelated SLC25 family mitochondrial ATP/ADP carrier, where lack of NMPs transport was assumed to occur because of reduced contact points at the binding site (22, 23, 42).

## Experimental procedures

### Materials

Common chemicals used in this work were of analytical grade and obtained from Sigma-Aldrich Co. A2252-Adenosine 5′-monophosphate monohydrate, ADP (A2754 >95% bacterial HPLC), dATP (100 mM 10297–117, Invitrogen), ATPɣ[-S] (Tetralithium salt 100 mM, Calbiochem 119123), GMP (disodium salt hydrate from yeast, ≥99%, G8377), GDP (sodium salt Type I, ≥96% (HPLC), G7127), UMP (disodium salt >99%, U6375), UDP (sodium salt from *Saccharomyces cerevisiae*, 95–100%, U4125), UDP-Glc (disodium salt hydrate *Saccharomyces cerevisiae* ≥98%, 94335), UDP-Galactose (disodium salt ≥97.0%, U4500), UDP-N-Acetylglucosamine (sodium salt ≥98%, U4375), sodium phosphate monobasic (S8282), galactose (G0625), ammonium sulfate (A4418 ), dextrose (D9434), and sodium pyrophosphate dibasic (71501). ATP, CTP, GTP, and UTP 100 mM (27–2025–01, >98% triphosphate) were from GE Healthcare Life Sciences. n-dodecyl-ß-D-maltopyranoside (D310), and n-octyl-ß-D-glucopyranoside (O311) were obtained from Anatrace Products, Affymetrix, and Soy L-alpha-Phosphatidylcholine, 95% (PC, 441601) were purchased from Avanti Polar Lipids. Yeast nitrogen base w/o amino acids w/o ammonium sulfate (4027-032) and Yeast Synthetic Drop-out Medium Supplements without uracil (4511-222) were obtained from Bio 101 System. [α^32^P]ATP(BLU003X250UC) and [ɤ^32^P]ATP(NEG035C005MC) were obtained from PerkinElmer. Nickel NTA (NTA-Ni) was purchased from Qiagen and Bio-Beads SM-2 Resin from BioRads.

### Molecular biology

Unless otherwise noted, standard molecular biology protocols were applied as described (44). The *Homo sapiens* *SLC35B1* gene (*hSLC35B1*) was amplified by PCR from pET22bUGTrel1 plasmid (kindly gifted by Prof. Hirschberg) using short DNA polymerase (Bioline) and primers hB1 for 5′-TCGACGGATTCT AGAACTAGTGGATCCCCCATGGCCTCTAGCAGCTCCCTG-3′ and hB1rev 5′- AAATTGACCTTGAAAATATAAATTTTCCCCGTGGGATGTCTTCTTAGCTC-3′. The resulting PCR product was cloned, into the vector p426GFP (25) digested with SmaI, by homologous recombination in *S. cerevisiae* FGY217 (kindly provided by Prof. Ljungdahl) and selected in plates containing -Ura media, as described (45). A single colony was grown in -Ura media and the plasmid p426h*SLC35B1*GFP was isolated. The identity of the cloned fragment was confirmed by DNA sequencing.

Mutants K117A, K120A, R276A, and K277A were generated using the primers K117Ar 5′-GCAGGATGCACCAAG-3′, K120Ar 5′-GATTGGCGCGCAGGA-3′, R276Ar 5′-GAACTTTGCAGTTGT-3′, and K277Ar 5′-GAAGAACGCTCGAGT-3′ by the megaprimer method in two rounds of PCR (46). In the first round, a fragment (megaprimer) containing the corresponding mutation was obtained using the reverse primers (K117Ar, K120Ar, R276Ar, or K277Ar) and primer hB1for. In a second PCR round, the purified megaprimer fragments, corresponding to each variant, was used in combination with the primer hB1rev to obtain the full-length products of each variant. The p426*hSLC35B1*GFP plasmid was used as a template. The resulting PCR products variants were cloned in p426GFP, as was described to obtain p426*hSLC35B1*GFP. The identity of the cloned fragments was confirmed by DNA sequencing after isolation of the corresponding plasmids.

### Yeast strain, transformation, and growth media

For the transformation of *S. cerevisiae* FGY217, a lithium acetate/polyethylene glycol method was used (47). Briefly, the cells were grown in complete media YPD (0.75 % yeast extract, 1.13 % peptone, and 2.2 % dextrose) and transformed with the corresponding plasmids. Then, the transformants were selected by their ability to grow at 28 °C in the absence of uracil (-Ura media) on plates containing 0.67% yeast nitrogen base with ammonium sulfate without amino acids, 0.08 % Yeast Synthetic Drop-out Medium Supplements without uracil, 2.0 % dextrose, and 2.0 % agar.

### Expression and purification of hSLC35B1 variants

Expression and purification procedures of *hSLC35B1 variants* were performed essentially as previously described (48). Briefly, a single colony of *S. cerevisiae* FGY217 transformed with p426hSLC35B1GFP plasmid was used to inoculate -Ura media and grown to an A600 = 2.0. This culture was used to inoculate the preinduction media containing 2% peptone, 1% yeast extract, and 0.1% dextrose and grow from an initial A600 = 0.1 to an A600 = 0.8. The expression of SLC35B1GFPHisx8 was initiated by adding 2% galactose and incubated for 36 h at 30 °C. The cells were collected by centrifugation 10 min at 3000*g*. Total membrane fractions were prepared by glass bead disruption of yeast cells in rupture buffer (50 mM Tris–HCl, 10% glycerol, 5 mM EDTA, 1 mM PMSF, pH 7.6 at 4 °C). Suspensions were centrifuged 10 min at 2000*g*, followed by centrifugation of the supernatants at 100,000*g* for 1 h. Resulting membrane pellets were resuspended in membrane buffer (50 mM Tris–HCl, 10% glycerol, 1 mM PMSF, pH 7.6 at 4 °C). The purification procedure was initiated by solubilization of membranes at 3 mg/ml with 1% of DDM and incubating during 1 h at 4 °C under mild agitation. Nonsolubilized material was separated by centrifugation at 100,000*g* for 30 min. The supernatant was isolated, 150 mM NaCl was added, and mixed with NTA-Ni and was incubated 3 h at 4 °C under mild agitation. The column with NTA-Ni was washed with 20 volumes of 50 mM Tris–HCl (pH 7.6 at 4 °C), 150 mM NaCl, 10% glycerol, 0.1% DDM, and 30 mM imidazole. Finally, hSLC35B1GFPHisx8 was eluted in the same buffer containing 200 mM imidazole. To eliminate imidazol, the preparation of purified hSLC35B1GFPHisx8 was concentrated and diluted in buffer 50 mM Tris–HCl (pH 7.60 at 4 °C), 150 mM NaCl, 10% glycerol, 0.1% DDM, using Merck Millipore Amicon Ultra-2.0 Centrifugal Filter Units MWCO 100,000. The concentration of the preparation (∼0.5-1 μM) was estimated based on the fluorescence of hSLC35B1GFP and purified GFP used as a standard (λ_ex_ 485- λ_em_ 514) (48).

### Analysis by SDS-PAGE

The integrity of hSLC35B1GFP during the purification procedure was analyzed by electrophoresis on 12% SDS polyacrylamide gel (49). Gels were scanned using a Storm 840 molecular imager (Amersham Biosciences) to identify GFP-fluorescence containing bands and subsequently stained with Coomassie blue (48).

### Transport assay in liposomes

Reconstitution of purified hSLC35B1GFP into phosphatidylcholine (PC) liposomes was performed as described before (25, 50). Briefly, 1 to 10 μl of purified hSLC35B1GFP preparations (0.5–1 μM) was mixed with extruded PC (ratio PC/protein 2000) in a medium containing 10 mM Tricine-KOH (pH 7.50 at RT), 50 mM potassium gluconate, 20% glycerol, and 50 mM octyl-β-glucoside, in the absence or presence of the indicated concentration of ligands. Detergent was removed by bio beads (Bio-Rad), and hSLC35B1GFP-liposomes were isolated by gel filtration using Sephadex G-50 (GE Healthcare). To estimate the incorporation of hSLC35B1GFP in the liposomes, GFP-fluorescence associated to the void volume was measured using purified GFP as a standard (λ_ex_ 485- λ_em_ 514).

To assay the transport activity under standard conditions, hSLC35B1GFP reconstituted in PC-liposomes was incubated with the indicated [α^32^P]ATP (or [ɤ^32^P]ATP) concentrations for 1 min at 37 °C in a medium containing 10 mM Tricine–KOH (pH 7.50 at 37 °C), 50 mM potassium gluconate, and 20% glycerol. The reaction was stopped by gel filtration using Sephadex G-50. The radioactivity in the void volume, corresponding to [α^32^P]ATP (or [ɤ^32^P]ATP) incorporated in the hSLC35B1GFP-liposomes, was measured by liquid scintillation counting.

Separate experiments were performed to verify the effective ATP concentration of ATP-loaded proteoliposomes. For that purpose, a luminometric technique was used to assess ATP content, and ^3^H-GMP was used as a marker of the effective trapped aqueous volume of proteoliposomes (see Supp. Fig. 1). Trapped ATP concentration was then calculated as the ratio of ATP content over trapped aqueous volume.

### Bioinformatics analysis

The primary amino acid sequences of SLC35 family proteins were analyzed using TOPCONS topology predictor (51). Sequence alignments were generated using Clustal Omega (52).

### Human SLC35B1 homology modeling

A homology model of hSLC35B1 was generated using the Phyre2 Protein Fold Recognition Server (http://www.sbg.bio.ic.ac.uk/phyre2) (53). The model built was selected on the basis of the structure of Vrg4 (PDB ID: 5OGE). Model quality was tested using the structure-validate Web server Molprobity (http://molprobity.biochem.duke.edu) (54). Two hundred ninety-seven of three hundred twenty-five residues (92% of the sequence of hSLC35B1) have been modeled with 100.0 % confidence by the single highest scoring template Vrg4.

### Data analysis

At least three independent preparations of purified hSLC35B1 were evaluated for each experiment. Rates of ATP uptake were determined as initial rates of [α^32^P]ATP (or [ɤ^32^P]ATP) uptake and expressed as values in pmol/mg prot./min or as % of maximal values under optimal conditions, as indicated in the legends. Kinetic parameters were calculated by nonlinear regression using the least square minimization function of calc (open office) and were expressed as the mean ± SEM.

## Data availability

All data are contained in the article and the supporting information file.

## Supporting information

This article contains supporting information (55, 56, 57, 58).

## Conflict of interest

The authors declare that they have no conflicts of interest with the contents of this article.
