# Supplementary material for: The broad range di- and tri-nucleotide exchanger SLC35B1 displays asymmetrical affinities for ATP transport across the ER membrane
Source: J Biol Chem. 2022 Jan 15;298(4):101537. doi: 10.1016/j.jbc.2021.101537 (PMC9010763; doi:10.1016/j.jbc.2021.101537)
Supplement: Supplemental Figures S1–S5 [file mmc1.pdf]

Supporting Information

ER-hSLC35B1 is a broad range di- and trinucleotide exchanger displaying asymmetrical apparent affinities for activating transport.

Pablo J. Schwarzbaum, Julieta Schachter, and Luis M. Bredeston

From the Departamento de Química Biológica-IQUIFIB, Facultad de Farmacia y Bioquímica Universidad de Buenos Aires-CONICET, , CABA, Argentina.

Figure S1

Figure S2

Figure S3

Figure S4

Figure S5

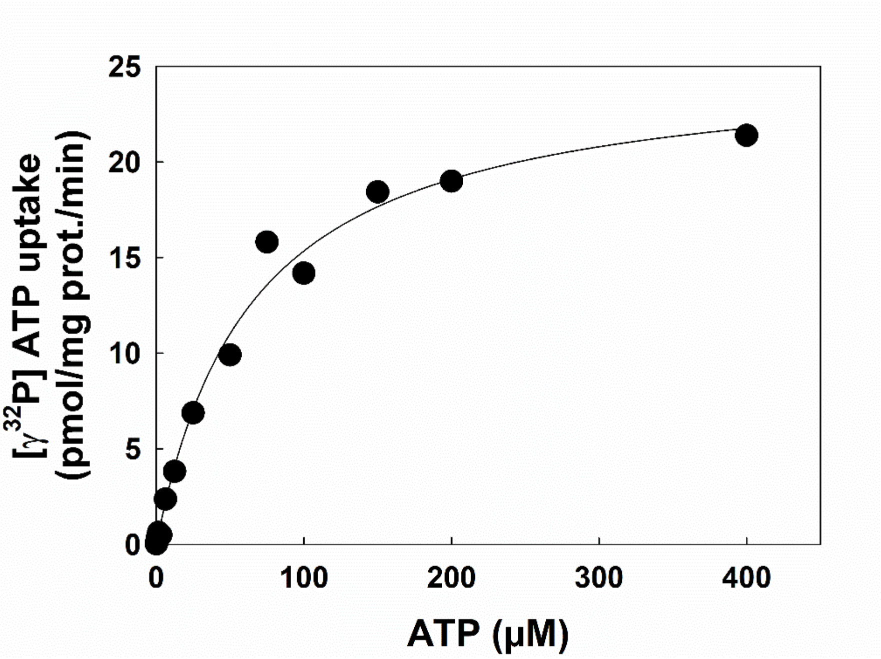

Figure S1. Dependence of [ɣ^{32}P]ATP uptake on external ATP concentration. hSLC35B1-liposomes were loaded with 10 mM ADP and incubated with the indicated [ɣ^{32}P]ATP concentrations for 1 min at 37ºC. Continuous lines represent the best fit of a single hyperbolic function to experimental data, with Vmax= 25.28±1.10 pmol/mg prot./min and $K_{0.5(\text{external ATP})}$ = 64.67±8.00 µM.

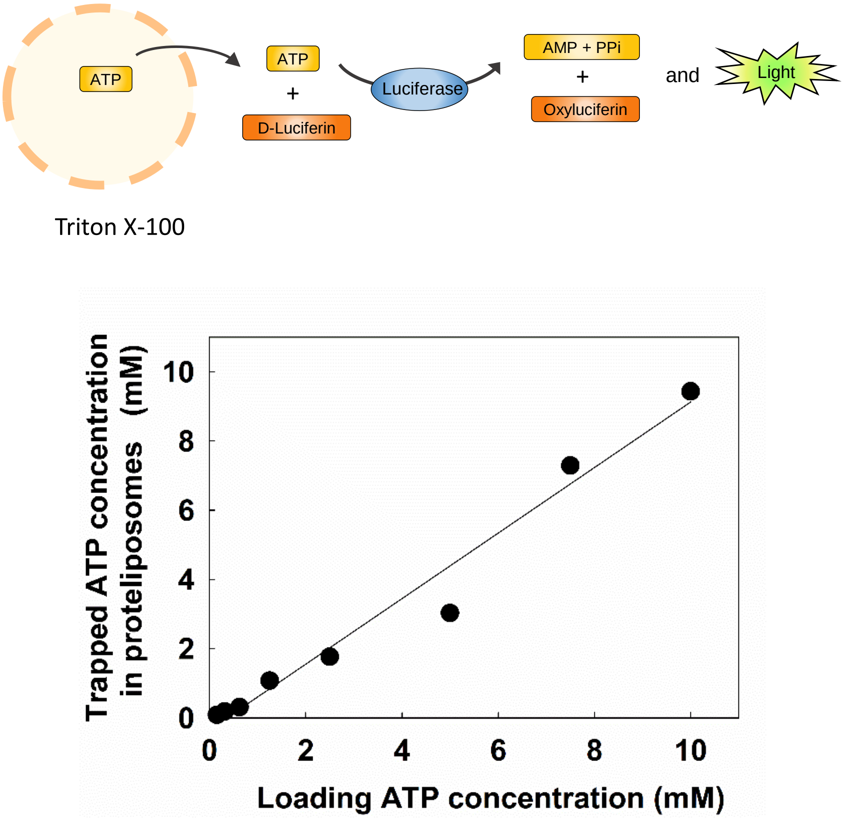

A

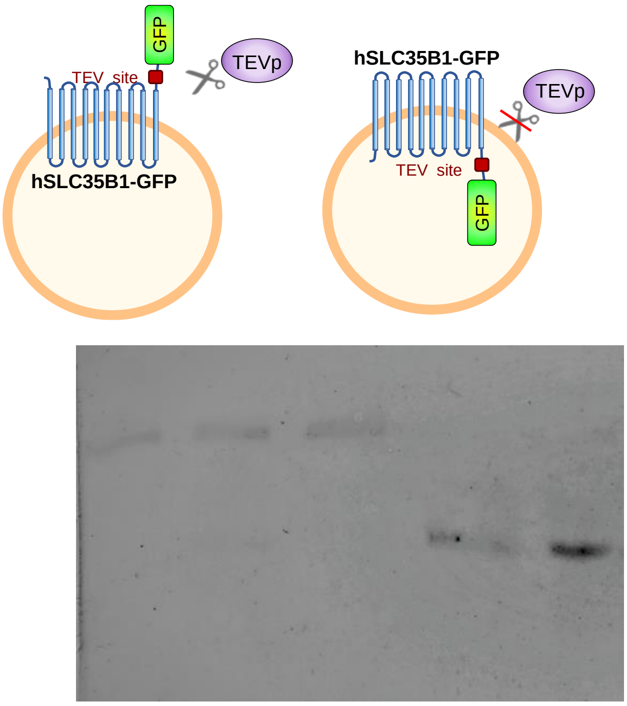

	1	2	3	4	5
**SLC35B1-GFP liposomes**	+	+	+	+	-
**TEV protease**	-	+	-	+	-
**Triton X-100**	-	-	+	+	-
**purified GFP**	-	-	-	-	+

B

Figure S2. Quality control of proteoliposome preparation. A. Effective concentration of ATP trapped inside proteoliposomes was measured by luminometry and remaining ^{3}H-GMP radiactivity. Aliquots of suspensions containing ATP-loaded proteoliposomes (10µl) were used to measure the total mass of ATP located in the lumen of proteoliposomes. On the other hand, ^{3}H-GMP loaded proteoliposomes were used to assess their captured aqueous volume. ATP concentrations trapped inside proteoliposomes were then calculated as the ratio between ATP mass and captured volume.

To assess ATP mass, a luciferin-luciferase luminometry technique was used, as described before (Montalbetti et al 2011). Briefly, experiments were run with proteoliposomes suspensions loaded with different ATP concentrations (20-100 nM). The scheme illustrates the experimental design. In the absence of detergent, a very low ATP signal was obtained. Then, suspensions were treated with 0.5% Triton X-100, and total ATP mass released in the assay medium was measured. We have shown before that, under the experimental conditions, ATP consumption by luciferase is negligible, so that this enzyme works as a quantitative ATP sensor (Leal Denis et al. 2013).

We used ^{3}H-GMP to measure the trapped aqueous volume, since it is impermeable to proteoliposomes and can be used as a marker of intravesicular volume. ^{3}H-GMP was incorporated into proteoliposomes using a similar procedure as described for the other nucleotides used in this study and the intravesicular volume estimated based on the remaining and initial radioactivity (Perkins et al., 1993).

Concentrations of ATP trapped inside liposomes were plotted vs ATP concentration added to the mixture during proteoliposome preparation. A linear function was fitted the data, with slope being 0.93 ± 0.083, $r^2=0.95$. Each point represents the average result of two independent experiments at a fixed ATP concentration added to the liposome mix preparation.

B. Orientation of hSLC35B1-GFP was evaluated based on the accessibility of the TEV protease to the TEV site between hSLC35B1 and GFP in the absence and presence of Triton X-100 (Islam et al., 2013). As depicted in the scheme, the TEV site can be located at the outside or inside surfaces of the liposome. Suspensions of hSLC35B1-GFP liposomes were incubated 6 hs at 30°C in the absence (lanes 1 and 2) and presence (lanes 3 and 4) of 0.3% of Triton X-100 with in the presence of 4 µM TEV (lanes 2 and 4) or in its absence (lanes 1 and 3). After incubation, the samples were mixed with loading buffer and protein separated by 12% SDS-PAGE electrophoresis. Purified GFP was run as a control (lane 5). Images of the fluorescence bands were obtained using ImageQuant LAS 500.

<u>References</u>

Islam, S. T., Eckford, P. D., Jones, M. L., Nugent, T., Bear, C. E., Vogel, C., and Lam, J. S. (2013) Proton-dependent gating and proton uptake by Wzx support O-antigen-subunit antiport across the bacterial inner membrane. mBio, **4**, e00678-13. https://doi.org/10.1128/mBio.00678-13

Leal Denis, M.F., Incicco, J.J., Espelt, M.V., Verstraeten, S.V., Pignataro, O.P., Lazarowski, E.R., and Schwarzbaum, P.J. (2013) Kinetics of extracellular ATP in mastoparan 7-activated human erythrocytes" Biochimica et Biophysica Acta - General Subjects. **1830**, 4692-4707

Montalbetti, N., Leal Denis, M.F., Pignataro, O.P., Kobatake, E., Lazarowski, E.R., and Schwarzbaum, P.J. (2011) Homeostasis of extracellular ATP in human erythrocytes. J. Biol. Chem. **286**, 38397-38407

Perkins, W.R. , Minchey, S.R., Ahl, P.L., and Janoff, A.S. (1993) The determination of liposome captured volume. Chem Phys Lipids **64**, 197-217

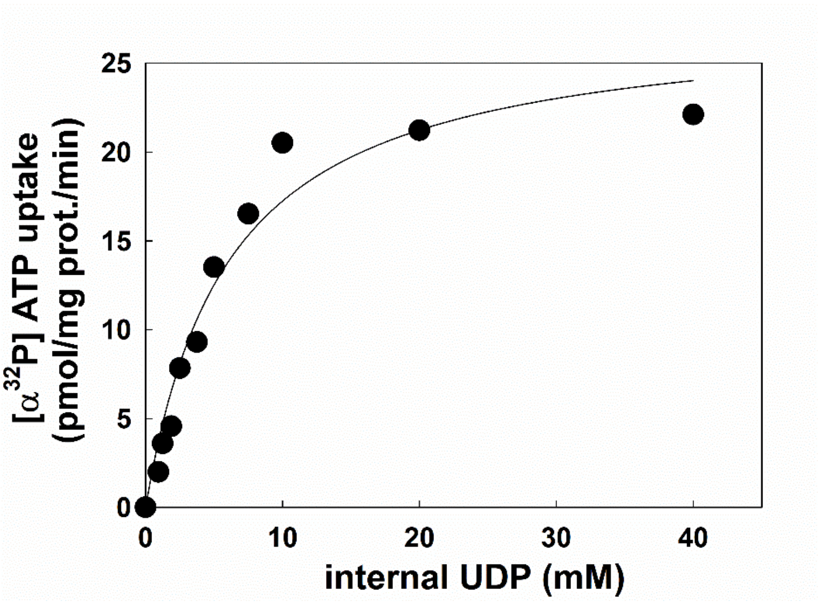

A

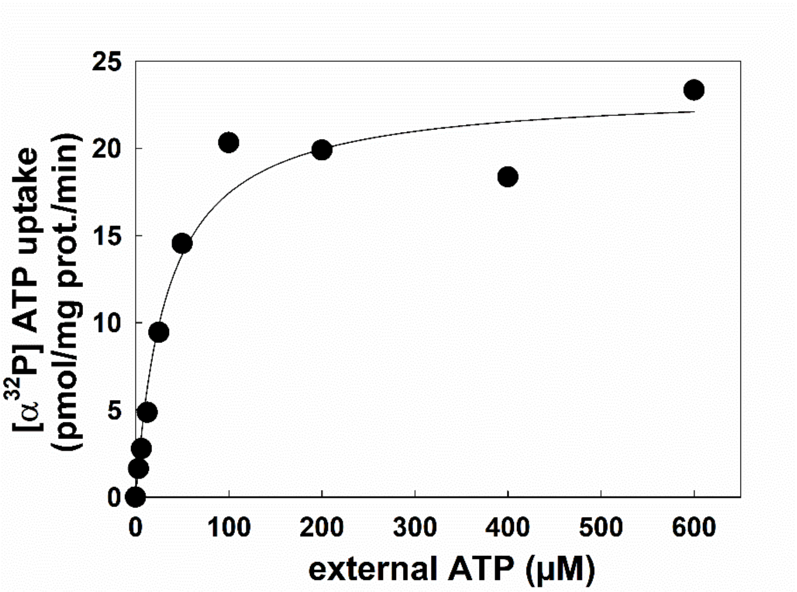

B

Figure S3. Effect of internal UDP on hSLC35B1-liposomes transport activity (A) Dependence of [α^{32}P]ATP uptake on internal UDP concentration. hSLC35B1-liposomes were loaded with the indicated UDP concentrations and incubated with 200 µM [α^{32}P]ATP 1 min at 37 °C. (**B**) Dependence of [α^{32}P]ATP uptake on external ATP concentration. hSLC35B1-liposomes were loaded with 20 mM UDP and incubated with the indicated [α^{32}P]ATP concentrations for 1 min at 37 °C. The reaction was stopped and the incorporated [α^{32}P]ATP to hSLC35B1-liposomes was measured. The continuous lines represent fitting of a single hyperbolic function to experimental data. The best-fit parameter values were: (A) Vmax: 27.64±2.14 pmol/mg prot./min and $K_{0.5(\text{internal UDP})}$: 6.03±1.20 mM, and (B) Vmax: 23.35±1.32 pmol/mg prot./min and $K_{0.5(\text{external ATP})}$ 33.99±7.53 µM.

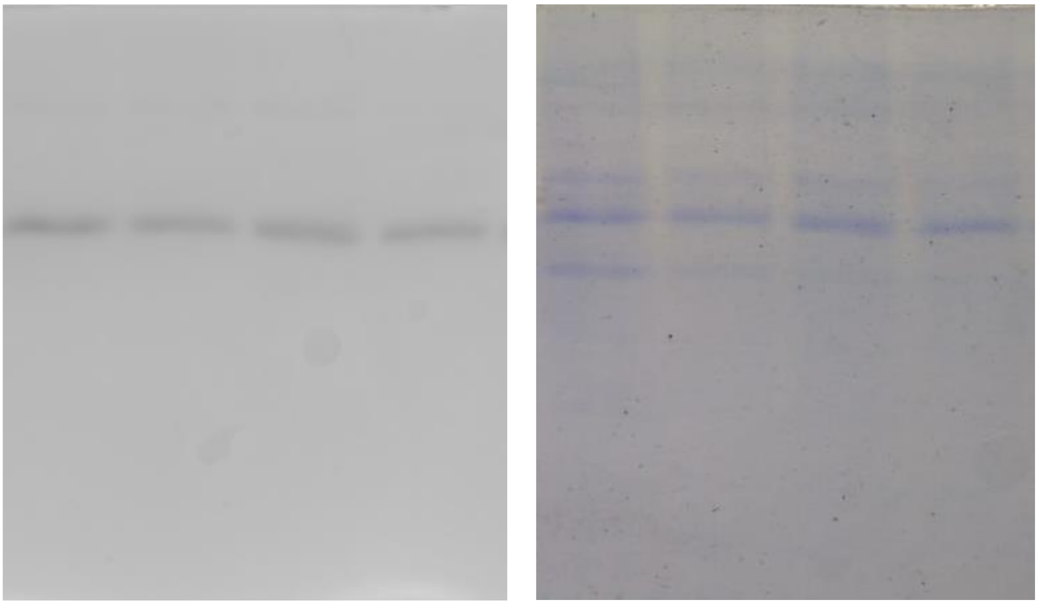

Figure S4. Analysis of purified hSLC35B1 variants. Proteins purified from yeast membranes expressing WT and the K117A, K120A, or K277A variants were separated by SDS-PAGE and GFP fusions visualized by in gel fluorescence and CB staining.

```
hSLC35B4      ------------------------------------------------------------   0
hSLC35B3      ------------------------------------MDLTQQAKDIQNI------TVQ--   16
hSLC35B2      MDARWWAVVVLAAFPSLGAGGETPEAPPESWTQLWFFRFVVNAAGYASFMVPGYLLVQYF   60
hSLC35B1      ------------------------------------------------------------   0


hSLC35B4      -----------------------------------------------------------M   1
hSLC35B3      -ETNKNNSESIECSKITMDLKFNNSRKYISITVPSKTQTMSPHIKSVDDVVVLGMNLSKF   75
hSLC35B2      RRKNYLETGRGLCFPLVKACVFGNEPKA-SDEVPLAPRTEA-------------AETTPM   106
hSLC35B1      ---------------------------------------MA-------------SSSSLV   8
                                                                         .

hSLC35B4      RPALAVGLVFAG---------CCSNVIFLELLARKHPGCGN------IVTFAQFLFIAVE   46
hSLC35B3      NKLTQFFICVAGVFVFYLIYGYLQELIFSVE---GFKSCGW------YLTLVQFAFYSIF   126
hSLC35B2      WQALKLLFCATGLQVSYLTWGVLQERVMTRSYGATATSPGERFTDSQFLVLMNRVLALIV   166
hSLC35B1      PDRLRLPLCFLGVFVCYFYYGILQEKITRGKYGEGAKQETFTFA--LTLVFIQCVINAVF   66
                   . :   *           .: :                     :.: :  :  :
                        TM1                                          TM2

hSLC35B4      GFLFEADL-GRKPPAIPIRYYAIMVTMFFTVSVVNNYALNLNIAMPLHMIFRSGSLIANM   105
hSLC35B3      GLIELQLIQDKR-RRIPGKTYMIIAFLTVGTMGLSNTSL-GYLNYPTQVIFKCCKLIPVM   184
hSLC35B2      AGLSCVLCKQPR-HGAPMYRYSFASLSNVLSSWCQYEAL-KFVSFPTQVLAKASKVIPVM   224
hSLC35B1      AKILIQFFDTARVDRTRSWLYAACSISYLGAMVSSNSAL-QFVNYPTQVLGKSCKPIPVM   125
              . :        :        *       .     .  :*   :  * ::: :. . *  *
                                       TM3                       TM4
hSLC35B4      ILGIIILKKRYSIFKYTSIALVSVGIFICTFMSAKQVTSQSSLSENDGFQAFVWWLLGIG   165
hSLC35B3      LGGVFIQGKRYNVADVSAAICMSLGLIWFTLADS---TTAPNFN-----------LTGVV   230
hSLC35B2      LMGKLVSRRSYEHWEYLTATLISIGVSMFLLSSGPEPRSSPATT-----------LSGLI   273
hSLC35B1      LLGVTLLKKKYPLAKYLCVLLIVAGVALFMYKPKKVVGIEEHTV-----------GYGEL   174
              : *  :  : *   .      :  *:                               *
                                 TM5
hSLC35B4      ALTFALLMSARMGIFQETLYKRFGKHSKEALFYNHALPLPGFVFLASDIYDHAVLFNKSE   225
hSLC35B3      LISLALCADAVIGNVQEKAMKLHNASNSEMVLYSYSI---GFVYILLGL------TCTSG   281
hSLC35B2      LLAGYIAFDSFTSNWQDA-LFAYKMSSVQMMFGVNFF---SCLFTVGSL------LEQGA   323
hSLC35B1      LLLLSLTLDGLTGVSQDHMRAHYQTGSNHMMLNINLW---STLLLGMGI------LFTGE   225
               :   :   .. .  *:     .   . . ::        . :    .:         .
                     TM6                                         TM7
hSLC35B4      LYEIPVIGVTLPIMWF-YLLMNIITQYVCIRGVFILTTECASLTVTLVVTLRKFVSLIFS   284
hSLC35B3      LGPAVTFCAKNPVRTYGYAFLFSLTGYFGISFVLALIKIFGALIAVTVTTGRKAMTIVLS   341
hSLC35B2      LLEGTRFMGRHSEFAA-HALLLSICSACGQLFIFYTIGQFGAAVFTIIMTLRQAFAILLS   382
hSLC35B1      LWEFLSFAERYPAIIY-NILLFGLTSALGQSFIFMTVVYFGPLTCSIITTTRKFFTILAS   284
              *     :            ::  :        ::      .      : * *: .::: *
                                     TM8                         TM9
hSLC35B4      ILYFQNPFTLWHWLGTLFVFIGTLMYTEVWNNLGTTKSEPQKDSKK--------N-----   331
hSLC35B3      FIFFAKPFTFQYVWSGLLVVLGIFLNV-YSKNMDKIRLPSLYDLINKSVEARKSRTLAQT   400
hSLC35B2      CLLYGHTVTVVGGLGVAVVFAALLLRV-YARGRLKQRGKKAVPVES-PV---------QK   431
hSLC35B1      VILFANPISPMQWVGTVLVFLGLGLDAKFGKGAKKTSH----------------------   322
               : : : .:     .  .*. .  : .   ..  .
                                   TM10
hSLC35B4      -     331
hSLC35B3      V     401
hSLC35B2      V     432
hSLC35B1      -     322
```

Figure S5. Conservation of charged residues of TM4 and TM9 on SLC35B-subfamily. Alignment of human SLC35B1, SLC35B2, SLC35B3, and SLC35B4 members of the SLC35B family. Transmembrane helices were depicted in bold. Conserved charged residues were labelled in yellow, and the C-terminal ER retention motif was labelled in red.
